# The Effect of Nicotine Dependence on Psychopathology in Patients with Schizophrenia

**DOI:** 10.1155/2015/730291

**Published:** 2015-04-28

**Authors:** Anne Yee, Nik Nasyrah Bt Nek Mohamed, Aili Hanim Binti Hashim, Huai Seng Loh, Manveen Kaur Harbajan Singh, Chong Guan Ng, S. T. Jambunathan

**Affiliations:** ^1^Department of Psychological Medicine, Faculty of Medicine Building, University of Malaya, 50603 Kuala Lumpur, Malaysia; ^2^Department of Psychiatry and Mental Health, Hospital Selayang, Lebuhraya Selayang-Kepong, 68100 Batu Caves, Selangor, Malaysia; ^3^Clinical Academic Unit (Family Medicine), Newcastle University Medicine Malaysia, No.1 Jalan Sarjana 1, Kota Ilmu, Educity@Iskandar, 79200 Nusajaya, Johor, Malaysia

## Abstract

*Introduction*. Our study aims to determine the prevalence of nicotine dependence and investigate the effect of nicotine dependence on psychopathology among schizophrenia patients. *Methods*. A cross-sectional study was carried out in an outpatient psychiatric clinic at a general hospital in Malaysia. 180 recruited subjects were administered the Malay version of Mini International Neuropsychiatric Interview (MINI), the Positive and Negative Symptom Scale (PANSS), and the Malay version of Fagerstrom Test for Nicotine Dependence (FTND-M) questionnaires. *Results*. The prevalence of nicotine dependence among the subjects was 38.1% (*n* = 69) and they were mainly composed of male gender, Malay ethnicity, being treated with atypical antipsychotics, and taking other illicit drugs or alcohol. Subjects with severe nicotine dependence scored less in the negative subscale of PANSS compared with the nonsmokers (*P* = 0.011). On performing the hierarchy multiple regressions, dependence status still significantly predicted negative scores after adjusting the confounders (*t* = −2.87, *P* = 0.005). *Conclusion*. The rate of nicotine use disorder among schizophrenia patients in this study is higher than that of the general population in Malaysia. The significant association between nicotine dependence and negative psychopathology symptoms will help the healthcare practitioners in their management of nicotine dependence among schizophrenia patients.

## 1. Introduction

Schizophrenia is a severe and disabling mental illness [1]. Persons with schizophrenia are at high risk of shorter life expectancy [2–4], due to the increased mortality related to circulatory and respiratory diseases, with chronic cigarette smoking being a major contributory factor [5, 6].

Worldwide statistics have shown that smoking contributes significantly to mortality [7, 8], with nicotine being more addictive than alcohol, marijuana, or cocaine [9]. Compared to the general healthy population, the prevalence of smoking is found to be much higher among people with psychotic disorders [10–12] and, among those suffering from schizophrenia, the occurrence of smoking and nicotine dependence is higher than that of both the general population and those with other mental illnesses [13–17]. Schizophrenic patients who smoke tend to have higher frequency of heavy smoking [8, 12], with rates ranging from 60% [16] to as high as 80% [18]. In addition, they are less likely to quit smoking [12, 19–22]. Hence, it is not surprising that smokers with psychiatric disorders suffer higher rates of morbidity and mortality secondary to smoking related illnesses [22, 23].

Smoking is the leading preventable cause of morbidity and premature mortality [24–26]. In Malaysia, one out of every five deaths is related to smoking. This is significant as smoking is the most important modifiable cause of premature death, responsible annually for an estimated 120,000 years of potential life loss [27]. The objective of this study is therefore to assess the prevalence of smoking and nicotine dependence among schizophrenic patients in a general hospital and to determine its associated factors, including the severity of illness.

## 2. Methods

180 patients were recruited from the outpatient psychiatric clinic in a general hospital from August to November, 2011, via purposive sampling method. The clinic had two follow-up sessions each week, with an average of 100–160 patients, and it covered a wide spectrum of psychiatric cases. It was estimated that 50–60% of the attendees met the diagnosis of schizophrenia. Inclusion criteria were patients who (a) met DSM-IV-TR [28] criteria for schizophrenia, (b) were at least 18 years of age, (c) provided informed consent, and (d) were able to understand and communicate in English or Bahasa Malaysia in order to complete the study measurements. Exclusion criteria were those who (a) had Axis I diagnosis other than schizophrenia or substance use disorder, (b) had an organic mental disorder, (c) had a diagnosis of mental retardation, (d) were experiencing unstable general medical conditions, and (e) were grossly psychotic and unable to cooperate with the interviewer. All patients who were identified as having schizophrenia in the clinic were approached. Written informed consent was obtained from patients who chose to participate in the study, after the discussion of study details with the researcher. 19 patients were excluded due to various reasons. The study was conducted in accordance with the Declaration of Helsinki and the National Medical Research Registry of the Ministry of Health and The Ministry of Health Medical Research Ethical Committee, which approved and monitored the study (Ethical committee reference no. NMRR-11-697-9166).

The study was conducted by a single interviewer who was trained in the use of the study instruments. A demographic and clinical data sheet was used to aid in the collection of the variables addressed in the study which, among others, included gender, ethnicity, medication use (typical or atypical antipsychotic), and history of substance use (lifetime use of alcohol, opioid, metamphetamine, cannabis, and ecstasy). This information was gathered via direct interview and/or review of patient's clinical records. The subjects were administered the Malay version of Mini International Neuropsychiatric Interview (MINI), the Positive and Negative Symptom Scale (PANSS) to rate the symptoms of schizophrenia [29], and the Malay version of Fagerstrom Test for Nicotine Dependence (FTND-M) [30]. The interview was completed with the subjects performing a breath test to measure the levels of carbon monoxide in their exhaled air, by using the simple handheld breath analyzer (piCO+* Smokerlyzer*). This instrument provided a direct measure of the carbon monoxide in parts per million (CO ppm). The MINI is a short structured diagnostic interview for researchers to accurately determine the presence of any psychiatric disorders according to DSM-IV or ICD-10 [31, 32]. It was administered in either English or in the Malay language, according to the patient's preference and main spoken language. Previous research has shown that 39.4% of smokers failed to meet the DSM-IV-TR criteria for nicotine dependence [33]. Hence, the FTND-M was used to assess the nicotine dependence among schizophrenia patients in this study. This easy-to-use self-report questionnaire is noninvasive, low-cost, and easy to score and has good validity and reliability for determining nicotine dependence among the smokers [30]. In our study, the patients were categorized as nicotine dependent if they had a score of more than two of that on the FTND-M [30]. Furthermore, all the nicotine-dependent patients were further classified as having high/severe dependence (FTND-M > 6) or mild-moderate dependence (FTND-M ≤ 6), as defined in previous studies [34, 35].

### 2.1. Statistical Analysis

Analyses of data were performed using the Statistical Package for Social Studies (SPSS) to generate the relevant descriptive epidemiological statistics. Differences between nicotine dependence and nonnicotine dependence on categorical variables were tested using either the chi-squared test or Fisher's exact test. To examine the relationship between nicotine dependence and psychopathology (PANSS), multivariate general linear model approach was used, with covaried gender, ethnicity, use of atypical antipsychotic medication, and comorbid use of illicit drug or alcohol, with familywise error multiple testing corrections where appropriate. A bootstrap analysis was conducted, using Preacher and Hayes' indirect macro for SPSS, to examine the indirect effect of nicotine dependence (independent variable) and psychopathology (dependent variable). For each analysis, 1000 random samples of the original size were taken from the obtained data, replacing each value as it was sampled. Statistical significance was evaluated at the <0.05 level using two-sided test.

## 3. Results

The study group comprised predominantly males (64.1%, *n* = 116) with the mean age of 41.5 years old (SD = 11.41). The current prevalence of nicotine dependence in the study subjects was found to be 38.1% (*n* = 69). The exhaled carbon monoxide in parts per million was correlated positively with FTND-M scores (Pearson's rho = 0.739, *P* < 0.01). The mean number of cigarettes smoked in the sample was 17 sticks per day. Among the subjects who were smoking, they have been smoking for a mean of 19.94 years and they have started smoking at the mean age of 19.7 years (SD 6.25). In 37% of the subjects, there was a positive family history of smoking. Sociodemographic characteristics and clinical details, according to smoking status, are shown in Table 1.

Multivariate analysis of covariance was used to examine the relationship between nonsmokers (*n* = 112, 61.9%), smokers with severe nicotine dependence (*n* = 21, 11.6%), mild-moderate nicotine dependence (*n* = 48, 26.5%), and PANSS scores, while controlling the gender, ethnicity, use of atypical antipsychotic medication, income, and comorbid using illicit drug or alcohol (Table 2). The nicotine dependence status was significantly associated with the PANSS scores (Wilk's lambda 0.898; *F*(2.37) = 2.78; *P* = 0.017). We conducted pairwise comparisons of the total and subscale PANSS scores between nonsmokers, those with mild dependence and those with severe dependence, using Bonferroni familywise error multiple testing corrections. Those with severe nicotine dependence scored less in the negative subscale compared to that of the nonsmokers (*P* = 0.015). On performing the hierarchy multiple regressions, dependence status still significantly predicted negative scores after adjusting the confounders (*t* = −2.87, *P* = 0.005).

## 4. Discussion

Based on all the literature reviewed, prevalence of smoking is found to be high among people with schizophrenia and our current study found a similar pattern. The prevalence of nicotine dependence found in this study was 38.1% (*n* = 69) with the subjects being more likely to be male and from the Malay ethnic group. They were also more likely to be taking illicit drugs or alcohol and were on atypical antipsychotic treatment. The findings of this study showed that subjects with severe nicotine dependence had less severity in negative symptoms compared to the nonnicotine dependent subjects.

The smoking rate of this study was much lower than it was reported in some studies done in the west [8, 16, 36] and among schizophrenic patients in a Chinese population [37]. The possible reason for the lower prevalence of nicotine use disorder in this study could be related to the lower prevalence of nicotine use among the Malaysian general population, estimated to be 23.1%, compared to other countries, which ranged from 27% to 43.3% [38].

There has been no study on ethnicity, though the National Health and Morbidity Survey 1996 found that Malay men were more likely to smoke [39]. Other studies have shown similar male preponderance [10, 37], concurrent hazardous use of alcohol and other illicit drugs [8, 11, 15], with studies suggesting that smoking is a reliable clue for other substance use and abuse [8] including alcohol [40, 41].

Smoking alters the level and effectiveness of medications in the blood, as it is hypothesized that nicotine interacts with many of the same central pathways thought to be abnormal in persons with schizophrenia [8, 36, 42]. Smoking also increases the metabolism of neuroleptics [43–45], with studies showing that individuals with schizophrenia and smoking tend to receive consistently higher doses of antipsychotics compared to nonsmokers [14, 46, 47]. The choice of pharmacological treatment is likely to have influence on smoking behavior [48], as earlier studies found that typical antipsychotics were associated with increased smoking in some individuals [49, 50] and they had greater difficulty to quit smoking [36]. Several reports have suggested that atypical antipsychotics, namely, clozapine, have helped in the reduction of smoking among schizophrenic smokers who have switched to this medication [51, 52]. In a small group of patients with schizophrenia or schizoaffective disorder, who were treated with transdermal nicotine patches and atypical or typical antipsychotic medications, George et al. [36] found a more favorable cessation rates among smokers who had received risperidone and olanzapine, compared to those on typical antipsychotics.

The literature is divided into smoking and symptoms of schizophrenia [10, 53]. In this present study, subjects who were severely nicotine dependent scored less in the PANSS negative symptoms. This finding seemed to be in agreement with other previous studies which showed that smokers had lower negative symptoms [6, 54–56]. A preclinical trial came up with evidence that nicotine affected several neurotransmitter systems, including dopamine, glutamate, and *γ*-aminobutyric acid (GABA), and certain neurocognitive deficits associated with these neurotransmitters improved after nicotine was administered in patients with schizophrenia. However, dopamine has been found to be the most implicated when the relationship between nicotine use and negative symptoms was studied [57]. By stimulating the release of dopamine in the nucleus accumbens and prefrontal cortex, nicotine reduces the negative symptoms of schizophrenia, explaining its use as a form of self-medication [58]. These positive effects could be an important mechanism that explains the comorbidity of schizophrenia and nicotine dependence [59].

Our study has several limitations that need to be highlighted. Firstly, as the information collection was done via self-reporting, it raised the possibility of underestimating the other substances. Secondly, it was not possible to identify a causal relation between the association of smoking and nicotine dependence and other variables because of the cross-sectional design of this study. Thirdly, there was no comparison made with a control group. Nevertheless, our study was able to prove that smoking is not only prevalent among the study subjects but reflects the severity of patients' illness.

## 5. Conclusion

The rate of nicotine use disorder among schizophrenia patients in this study is higher than that of the general population in Malaysia. Although causal links cannot be inferred, our study found a significant association between nicotine dependence and negative psychopathology symptoms, which will be an added value for the healthcare practitioners in their management of nicotine dependence among schizophrenic patients.

## Figures and Tables

**Table 1:** Sociodemographic characteristics and clinical features according to nicotine dependence.

Characteristics	Nicotine dependence	Nonnicotine dependence	Statistic	*P*	OR/mean difference (95% CI)
	*n* = 69	*n* = 112			
Age, years: mean (s.d.)	39.6 (10.8)	42.6 (11.6)		0.087	
Male, *n* (%)	68 (98.6)	48 (42.9)	*χ* ^2^ = 57.5	^**^ <0.001	90.6 (12.1–676)
Ethnicity, *n* (%)					
Malay	41 (49.4)	42 (37.5)			
Chinese	20 (29)	50 (44.6)	*χ* ^2^ = 8.3	^**^ <0.001	
Indian	8 (11.6)	20 (17.90)			
Duration of illness, years: mean (s.d.)	13.9 (10.4)	15.6 (9.8)		0.287	
Atypical antipsychotic, *n* (%)	46 (66.7)	53 (53.5)	*χ* ^2^ = 6.4	^**^0.014	2.23 (1.19–4.15)
Chlorpromazine equivalents (mg), mean (s.d.)	276.8 (190.6)	291.1 (487.0)		0.816	
Taking illicit drugs/alcohol, *n* (%)	19 (27.5)	2 (1.8)	*χ* ^2^ = 27.6	^**^ <0.01	0.05 (0.01–0.21)
Fagerstrom Test for Nicotine Dependence* *score, mean (s.d.)	4.16 (2.36)	—			
CO ppm (s.d)	13.91 (7.16)	2.22 (0.65)		^**^ <0.01	11.69 (9.97–13.42)
Marital status, *n* (%)					
Single	50 (72.5)	72 (64.3)		0.22	
Divorced	2 (2.9)	1 (0.9)			
Married	17 (24.6)	39 (34.8)			
Employment, *n* (%)					
Employed	41 (59.4)	55 (49.1)		0.22	
Unemployed	28 (40.6)	57 (50.9)			
Total income, *n* (%)					
≤RM500	43 (62.3)	92 (82.1)		^*^0.014	11.35 (0.011–0.017)
RM501–1000	15 (21.7)	15 (13.4)			
RM1001–2000	6 (8.7)	2 (1.8)			
RM2001–3000	4 (4.8)	3 (2.7)			
>RM3000	1 (1.4)	0			
Education level, *n* (%)					
Primary	10 (14.5)	15 (13.4)			
Secondary	53 (76.8)	75 (67)		0.22	
College/university	6 (8.7)	21 (18.8)			
No education	0	1 (0.9)			

^*^ *P* < 0.05, ^**^ *P* < 0.01, s.d. = standard deviation, OR = odds ratio, CI = confidence interval, and CO ppm = carbon monoxide in parts per million.

**Table 2:** Hierarchical multiple regression between smoking severity and PANSS scores.

	Nonsmoker	Mild-moderate nicotine dependence	Severe nicotine dependence	N − M Mean difference	N − S Mean difference	M − S Mean difference
	*n* = 112	*n* = 48	*n* = 21			
Total PANSS score, mean, ^a^mean (s.d)	49.13	51.56	51.43	0.62	1.16	0.54
	50.33^a^ (10.28)	49.71^a^ (12.21)	49.17^a^ (9.22)	(*P* = 0.98)	(*P* = 0.95)	(*P* = 0.97)

Positive subscale score, mean, ^a^mean (s.d)	8.54	9.65	9.76	−0.09	−0.04	0.05
	8.94^a^ (2.64)	9.04^a^ (3.09)	8.99^a^ (3.93)	(*P* = 0.88)	(*P* = 0.95)	(*P* = 0.98)

Negative subscale score, mean, ^a^mean (s.d)	18.01	17.13	14.67	1.89	4.54	2.64
	18.41^a^ (5.67)	16.52^a^ (6.45)	13.87^a^ (4.48)	(*P* = 0.26)	(^*^ *P* = 0.011)	(*P* = 0.06)

General psychopathology subscale score, mean, ^a^mean (s.d)	22.70	24.02	24.14	0.50	0.65	0.14
	23.42^a^ (4.13)	22.92^a^ (6.27)	22.77^a^ (7.07)	(*P* = 1)	(*P* = 1)	(*P* = 1)

N, nonsmoker; M, mild-moderate nicotine dependence; S, severe nicotine dependence; and PANSS = Positive and Negative Syndrome Scale.

^*^ *P* < 0.05, ^**^ *P* < 0.01, s.d = standard deviation.

^a^Multivariate analysis of covariance with PANSS total score and subscale scores as dependent variables; covariates appearing in the model are evaluated at the following values: gender = 1.36, ethnicity = 1.70, drug and alcohol abuse = 1.12, income = 1.25, and atypical antipsychotic = 1.55. These tests are based on the linearly independent pairwise comparisons among the estimated marginal means and adjusted for multiple comparisons using Bonferroni familywise error correction.
